# Weedy Rice as a Novel Gene Resource: A Genome-Wide Association Study of Anthocyanin Biosynthesis and an Evaluation of Nutritional Quality

**DOI:** 10.3389/fpls.2020.00878

**Published:** 2020-06-11

**Authors:** Wenjia Wang, Minghui Zhao, Guangchen Zhang, Zimeng Liu, Yuchen Hua, Xingtian Jia, Jiayu Song, Dianrong Ma, Jian Sun

**Affiliations:** Rice Research Institute, Shenyang Agricultural University, Shenyang, China

**Keywords:** weedy rice, genome-wide association study, anthocyanin biosynthesis, nutritional quality, pericarp color

## Abstract

The pericarp color of rice grains is an important agronomic trait affected by domestication, and the color pigment, anthocyanin, is one of the key determinants of rice nutritional quality. Weedy rice, also called red rice because its pericarp is often red, may be a novel gene resource for the development of new rice. However, the genetic basis and nutritional quality of anthocyanin are poorly known. In this study, we used a genome-wide association study (GWAS) to find novel and specific QTLs of red pericarp in weedy rice. The known key gene site of red pericarp *Rc* was detected as the common genetic basis of both weedy and cultivated rice, and another 13 associated signals of pericarp color that were identified may contribute specifically to weedy rice pericarp color. We then nominated three pericarp color genes that may contribute to weedy rice divergence from cultivated rice based on selection sweep analysis. After clarifying the distribution and growth dynamics of pigment in weedy rice caryopsis, we compared its nutritional quality with cultivated rice. We found that sampled weedy rice pericarps had much greater quantities of anthocyanin, beneficial trace elements, free amino acids, and unsaturated fatty acids than the cultivated rice. In conclusion, the gene resources and novel genetic systems of rice anthocyanin biosynthesis explored in this study are of great value for the development of nutritious, high anthocyanin content rice.

## Introduction

Weedy rice (*Oryza sativa* f. *spontanea*) refers to rice plants that grow in rice fields or in surrounding fields as a weed. Weedy rice is spread throughout the rice paddy system worldwide, with temperate and subtropical areas being the hardest hit (Brespatry et al., 2001; Tai, 2002; Vaughan et al., 2017). Currently, research on the origin and evolution of weedy rice has made some progress, however, there is no definite conclusion about the origin of weedy rice (Li L. F. et al., 2017; Qiu et al., 2017; Sun et al., 2019).

In general, the morphological characteristics of weedy rice fall between the wild rice species (*Oryza rufipogon*) and cultivated rice (*Oryza sativa* L.) (Ma et al., 2008). Weedy rice has distinctive biological characteristics such as a short growth period, black hull, strong granulation, long dormancy period, long awning, and red pericarp (Chen et al., 2004; Pipatpongpinyo et al., 2019). Because most weedy rice has a colored hull and red pericarp, it is often called red rice (Ma et al., 2008). The layers of weedy rice caryopsis, from the outside to the inside, are pericarp, seed coat, nucellus, aleurone layer, and endosperm (Sellappan et al., 2009; Juliano and Tuaño, 2019). The colored pericarp is an important feature distinguishing it from ordinary cultivated rice (Cui et al., 2016).

Brown rice refers to the caryopsis after the rice husk is removed, and the pericarp, seed coat, and nucellus are intact. Milled rice refers to rice grains with pericarps and seed coats removed. Colored rice such as red rice, gold rice, and black rice, refers to brown rice with colored pericarp (Kim et al., 2008), and the caryopsis color is mainly due to the accumulation of anthocyanins in the pericarp and seed coat (Liu et al., 2011). Colored rice contains high levels of anthocyanins and proanthocyanidins, which have strong antioxidant properties (Lei et al., 2006). Some studies have shown that rice color is related to its nutritional quality. For example, brown rice is not only rich in protein, amino acids, vitamins, vegetable fats, and trace elements such as Ca, Fe, Zn, and Se, it is also rich in biologically active substances such as flavonoids that have anti-oxidation, anti-tumor, free radical-scavenging, and hypoglycemic effects (Gu, 1992; Meng et al., 2005; Guo et al., 2011; Yue-Ting et al., 2016).

With recent developments in genetic analysis, some molecular mechanisms regulating rice pigmentation are well known. For instance the red color of pericarp is affected by the interaction of two genes, *Rc* and *Rd*, and the purple color of pericarp is controlled by the joint action of genes *Pb* and *Pp* (Furukawa et al., 2007; Rahman et al., 2013). Furukawa et al. (2007) found that the 14 bp deletion of the coding region of the *Rc* gene caused the loss of *Rc* gene function, resulting in a white pericarp. Two SNP mutations in the coding region of the *Rd* gene caused early termination of translation, resulting in the loss of *Rd* function (Furukawa et al., 2007; Sweeney et al., 2007). Studies have shown that the pericarp color of the weedy rice in southern China is mainly regulated by the *Rc* gene. The same results were found in American weedy rice (Yang et al., 2009; Gross et al., 2010). As a companion weed of cultivated rice, weedy rice infests rice fields worldwide and is believed to have multiple evolutionary origins from distinct ancestors. Thus whether other genes besides the four known ones regulate its anthocyanin content is still unknown.

The pericarp color is an important agronomic trait affected by domestication, and the color pigment, anthocyanin, is one of the important determinants of rice nutritional quality (Wang et al., 2018). Weedy rice may be a novel gene resource for the development of new rice (Sun et al., 2013). However, the genetic basis and nutritional quality of anthocyanin are poorly known in weedy rice. Therefore, we used genome-wide association analysis to explore the main sites regulating the color of weedy rice pericarp. A follow-up study was carried out on the locations and timing of anthocyanin deposition in weedy rice with different pericarp colors. Also, nutritional quality was measured and compared between cultivated and weedy rice.

## Materials and Methods

### Materials

We collected 297 rice samples of six subgroups, including 46 weedy rice from Asian high latitudes (*WRAH*) and 14 middle-latitudes (*WRSC*), 69 temperate *japonica*, 12 tropical *japonica*, 145 *indica* and 11 *Aus*. The samples were divided into two sets for genome-wide association analysis (GWAS): set 1 for the red pericarp from weedy rice samples and set 2 the genetic background for the red pericarp from cultivar samples. All rice samples were maintained and cultivated in the germplasm resources field of Shenyang Agricultural University, China.

In order to further understand the production and distribution of weedy rice pericarp pigments, the typical red pericarp weedy rice WR07-14, WR07-47, WR03-32, WR07-141, WR07-142 and WR03-29 and white pericarp cultivated rice Shennong265, Akihikari, Nipponbare, Qishanzhan were selected from a GWAS panel for the observation of pigment components and their deposition process and for a nutrient quality analysis.

### Methods

#### Genome-Wide Association Study

For GWAS, the phenotype value of pericarp color was defined as one of four levels: 0 for white, 1 for orange, 2 for red, 3 for dark red. Short Oligonucleotide Alignment Program (SOAP) software (Li et al., 2008) was used to aligning the reads of each sample with the reference genome IRGSP v1.0 (Li et al., 2008). The reads that were successfully and uniquely aligned with the reference genome at both ends were considered reliable and used to define SLAF tags. We called population SNPs based on the corresponding genome-wide SLAF tags. A total of 122,777 unimputed SNPs with a minor allele frequency > 0.05 and integrity > 0.5 for evolutionary study have been reported in our previous study (Sun et al., 2019).

In the present studies, we performed imputation to fill the missing genotype for the 122,777 unimputed SNPs by using the k-nearest neighbor algorithm (KNN) model in two sets of GWAS populations (Roberts et al., 2007; Huang et al., 2010). Consequently, the quality of this 122,777 SNPs was improved with integrity > 0.9. Genome-wide association analyses were conducted using a compressed MLM model that could effectively reduce the false positives. The equation of the compressed MLM model is *y* = *X*α + *P*β + *K*μ + *e*, in which y is phenotype, *X* is genotype, *P* is population structure matrix (*Q* matrix), and *K* is the kinship matrix. The *P* matrix was built from the top five principal components for population structure correction. The *K* matrix was built from the matrix of simple matching coefficients. The analyses were performed using TASSEL 5 software (Bradbury et al., 2007). The threshold of significant *P*-value was determined based on Bonferroni correction method. In order to increase the possibility for overlapping the selection signal, we lowered the threshold of *P*-value by an order of magnitude from the Bonferroni correction threshold. Both thresholds of the significant *P*-value were used in the present study.

#### Selection Sweep Analysis and Candidate Gene Nomination

In this study, red pericarp rice (including landrace and weedy rice) were considered as the unimproved rice population, and modern cultivars with white pericarp were considered as the improved population. Nucleotide diversity (π) and Tajima’s *D* were obtained for 500 kb sliding windows between unimproved and improved populations by using vcftools 1.0.3 software (Danecek et al., 2011). Then the selection sweeps were defined by π ratio (π_unimproved population_/π_improved population_) and Tajima’s *D* in per 500 kb genomic slide windows and both selection signals were represented by heat maps. The cut-off of the π ratio for defining the strong selection genomic regions was set at 2.5, and the selected signal defined by negative value of Tajima’s *D* was as a secondary reference. We then nominated the candidate genes according to key motifs that related to anthocyanin synthesis from the Rice Genome Annotation Project^1^. We also searched nutrient-related genes around the genomic regions of GWAS peaks.

#### Developmental Dynamics of Pericarp Color and the Determination of Pigment Distribution

The weedy rice that we used to observe the development of pericarp color were sampled every 2 days after flowering. The caryopsis to be used for hand-sliced sections were sampled every week after flowering. A digital SLR camera (Canon 550D) was used to record pericarp color and pigment distribution (Liu et al., 2011).

We further sampled the pericarp, seed coat, and endosperm of weedy rice 1, 2, and 3 weeks after flowering respectively in order to study the distributions of their pigments. Referring to the method of Yu et al. (2010) we dissected these parts by paraffin section. The sample was dehulled and fixed with FAA fixative, and then dehydration, transparency, waxing, embedding, slicing, dipping, dewaxing and rehydration, dyeing, and sealing of the sample were carried out in sequence (Dai et al., 2009; Yu et al., 2010). The histological structure of the weedy rice caryopsis was recorded using a stereo microscope (Zeiss Lumar. V12).

#### Determination of Pigment Composition and Content

We collected seeds of each sample (WR07-14, WR07-32, WR07-47 and WR07-141) at 5, 10, 15, 20, and 25 days after flowering, and we threshed, shelled, and ground the rice into flour. The total content of anthocyanins was determined by the pH differential method (Li et al., 2014). The components of the pigment in the mature weedy rice were identified through High Performance Liquid Chromatography-Mass Spectrometer (HPLC-MS). The column temperature was set to 35°C. The binary mobile phase consists of a formic acid-ammonium formate solution. The mobile phase gradient decreased from 99% to 60%. The flow rate was 0.3 mL/min (Li R. et al., 2017).

#### Determination of Functional Nutrient Quality

Metal element concentrations in the pericarp of weedy rice and control samples were measured by using inorganic mass spectrometry (Gross, 2017). The amino acid concentrations were determined by chromatography using the Hitachi L8800 automatic amino acid analyzer (Hua et al., 2016). The fat was separated by soxhlet extraction for methyl esterification and then analyzed by using Gas chromatography–mass spectrometry (Agilent Technologies 7890A GC System and Agilent Technologies 240 Ion Trap). Resistant starch concentration was measured using the Shanghai Rongsheng Biotechnology Elisa kit.

### Statistical Analyses

Statistical analyses for the comparison of phenotypic values ANOVA were carried out with the statistical software IBM SPSS 2.00 (IBM Crop, Armonk, NY, United States). The level of significance taken as *P* < 0.05.

## Results

### Genome Wide Association Study of Weedy Rice Pericarp Color

**FIGURE 1:**
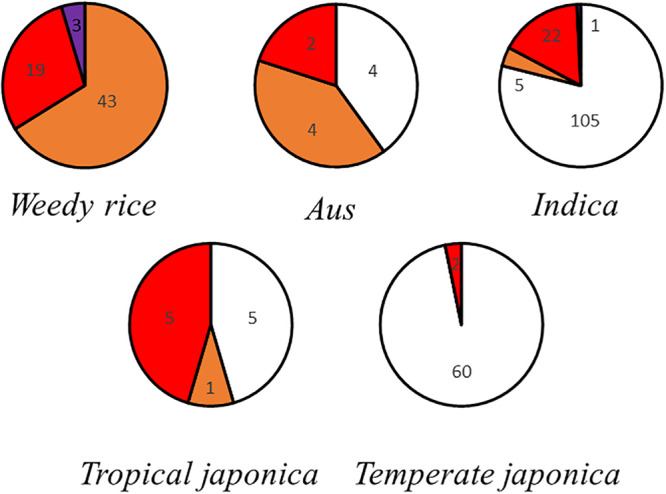
Pericarp color grading statistics. Each circle represents a subcategory of rice, and the area occupied by each color represents the proportion of rice seeds of each pericarp color.

The red pericarp phenotype was observed in all subgroups (Supplementary Figure S1). All weedy rice has colored pericarps, and some of these are extremely dark. *Aus*, *indica*, and tropical *japonica* have red pericarps and white pericarps. Most temperate *japonica* have white pericarps, and a few have red pericarps (Figure 1).

**FIGURE 2:**
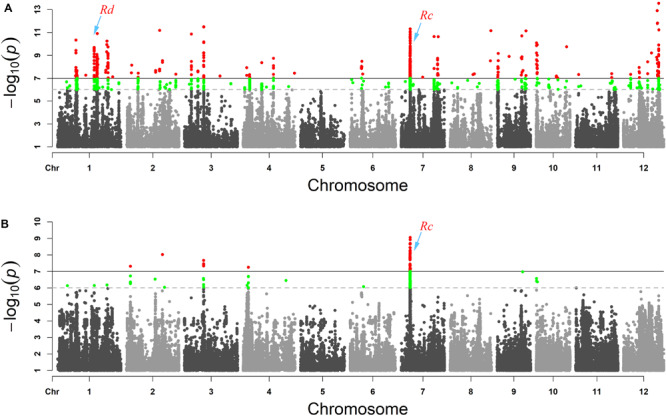
GWAS results presented as Manhattan plot for **(A)** red pericarp weedy rice and (set 1) **(B)** red pericarp cultivated rice (set 2). The red area in the Manhattan map is the response site associated with red pericarp. The known pericarp color genes *Rd* and *Rc* are labeled.

*WRAH* is a branch of temperate *japonica*, and *WRSC* is grouped with *indica* as reported in our previous research (Sun et al., 2019). In addition, the phenotypic variation of pericarp color was observed in all subgroups and did not correspond with subgroup differentiation. For these reasons, the false positives of GWAS due to population differentiation may have little impact, and we conducted GWAS across the six subgroups. In the compressed MLM of set 1 (weedy rice), we identified 13 association signals of pericarp color (*P* < 1 × 10^–7^), and in set 2 (cultivated rice), we identified four signals with clear peaks (*P* < 1 × 10^–7^) (Figure 2). The results showed that the genetic basis of pericarp color had both commonalities and differences between weedy rice and cultivated rice. The genetic basis of the pericarp color of weedy rice was more complex than that of the cultivated rice, which is reflected in the greater number of significant genomic association peak signals (Figure 2). Finally, we nominated five candidate genes according to the annotation information based on the Rice Genome Annotation Project (Table 1). The known key gene site of red pericarp *Rc* (Furukawa et al., 2007) was detected in both GWAS panels with significant peak signals. We also found that the association signals of red-pericarp synergistic-gene *Rd* could be detected in the weedy rice GWAS panel. The association signals of purple rice genes *Pp* and *Pb* could not be detected in either weedy rice or cultivated rice GWAS panels.

**TABLE 1:** GWAS peak selection sweep and candidate genes.

**Peak *P* value position**	***P* value**	**Genomic interval of selection sweep**	**π unimproved/ π improved**	**Tajima’s *D*** **value**		**Selection intensity**	**Pericarp color Candidate gene**	**Gene description**
				**Unimproved population**	**Improved population**			
Chr1:12490628	4.69E−11	12316796-12821546	2.12	3.21	–0.26	Weak		
Chr1:24954656	1.3E−09	24900449-25222998	1.87	3.11	0.09	Weak	LOC_Os01g43700.1(*Rd*)	Cytochrome P450 72A1, putative, expressed
Chr1:33719449	5.7E−11	33334269-33780964	2.42	3.04	–0.78	Weak	LOC_Os01g58950.1	Cytochrome P450, putative, expressed
Chr3:4655686	1.38E−11	4045839-4658179	2.78	2.6	–1.7	Strong	LOC_Os03g08930	Basic helix-loop-helix dimerisation region bHLH domain containing protein;
Chr3:9158937	1.12E−07	9326824-9810369	3.48	2.8	–0.79	Strong		
Chr3:13155280	3.11E−12	12620491-13044531	3.83	1.88	–1.45	Strong		
Chr4:12679715	3.22E−07	12320456-13135109	2.39	2.41	–0.97	Weak		
Chr4:20685858	1.82E−09	21337500-21848954	2.4	0.9	–1.3	Weak		
Chr6:8148068	3.41E−09	7070518-7601689	3.20	3.1	–1.2	Strong		
Chr7:6197350	4.88E−12	5536887-6172772	4.09	1.44	–0.78	Strong	LOC_Os07g11020 (*Rc*)	Pericarp color; red pericarp
Chr9:656027	6.98E−10	6084684-6583948	2.62	1.72	–1.46	Strong		
Chr10:296002	8.23E−11	2605942-3053511	1.61	1.94	–0.19	Weak		
Chr12:23036595	1.23E−13	22936893-23280883	3.16	0.56	–1.76	Strong	LOC_Os12g37419	Cytochrome c oxidase polypeptide Vc, putative, expressed 6

*Chr represents a chromosome.*

### Selection Sweep Analysis and Candidate Genes

**FIGURE 3:**
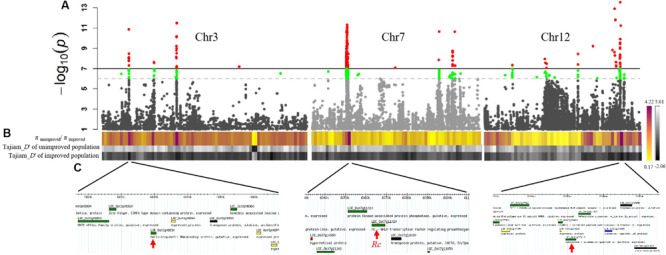
Selection sweep analysis and candidate genes for weedy rice pericarp color. **(A)** GWAS results of weedy rice pericarp color in Chr3 Chr7 and Chr12 presented as Manhattan plot. **(B)** The upper colored line in the figure is π unimproved population/π per 500 kb genomic window improved population. Red indicates the *P* value above the threshold. *P*-values determined based on Bonferroni correction method. In order to increase the possibility of overlapping with the selection signal, we reduced *P* by an order of magnitude based on the Bonferroni correction, which is indicated by the green peak. Red regions indicates the strong selection, whereas the green regions indicates weak selection. The middle and lower grayscale lines are Tajima’s *D* of the unimproved population and Tajima’s *D* of the improved population, respectively. The darker the color, the greater the intensity of the selection. **(C)** For each response peak, the chromosomal locations of the candidate genes for weedy rice pericarp color are shown.

Selection sweep analyses were conducted in the “unimproved” red pericarp population and in the “improved” cultivated white pericarp population. The selection sweep parameter of π_unimproved population_/π_improved population_ per 500 kb genomic slide windows were highlighted from yellow (0.17) to magenta (4.22) (Figure 3A). Another artificial selection parameter Tajima’s *D* represented by a black and white heat map, supported the selection sweep to define the selection genomic regions (Figure 3B). In order to nominate candidate genes that may contribute to the red pericarp color of weedy rice, we considered whether the GWAS peaks were covered by the selection signal. Finally, seven GWAS peaks with the strong selection signal were detected, of which the positions of known red pericarp gene *Rc* and two new candidate genes, *LOC_Os03g08930* and *LOC_Os12g37419*, were overlapped (Figure 3C and Table 1). In addition, a Glutelin-family-protein gene *Os03g0188500* (without MSU ID) was linked with *LOC_Os03g08930*, which may together contribute to grain quality and nutrition. Although the GWAS peak related *Rd* gene was detected, its selection signal was weak. The genome-wide GWAS peaks combined with the selection sweep were shown in Supplementary Figure S2.

### Developmental Dynamics of Weedy Rice Pericarp Color

**FIGURE 4:**
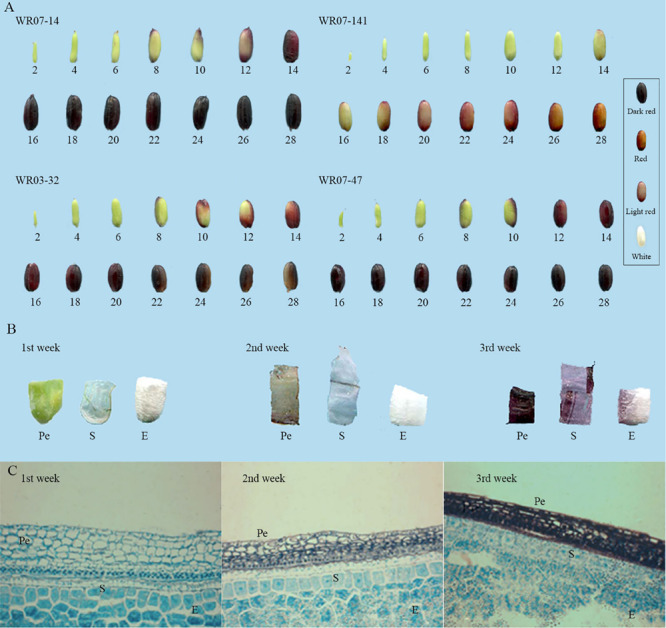
Changes through time in the appearance of the pericarp and pigmentation sites of typical weedy rice. **(A)** Developmental changes in caryopsis appearance in different colors of weedy rice. Numbers represent the days after flowering. **(B)** Pigmentation during weeks 1–3 after flowering (Pe, pericarp; S, seed coat; E, endosperm; weedy rice WR07-14 shown). **(C)** Pigment deposition in cells during weeks 1–3 after flowering (weedy rice WR07-14 shown).

The typical red pericarp weedy rice WR07-14, WR07-47, WR03-32, WR07-141, were selected from the GWAS panel for the observation of pigment components and the deposition process. The pericarp color of all sampled weedy rice was green in the early stage of development (0–5 days after flowering). The pigment began to deposit on both ends of the caryopsis (6–8 days after flowering), accumulating along the vascular bundle at the back of the caryopsis (9–15 days after flowering), and finally occurring throughout the entire caryopsis (16–18 days after flowering). When the seeds were mature, the pigmented pericarps were slightly faded (26–28 days after flowering) (Figure 4A).

We dissected the weedy rice WR07-14 caryopsis, including pericarp, seed coat, and endosperm (including aleurone layer) at different stages of development. At 1–7 days after flowering, the caryopsis was green, the seed coat was transparent, and the endosperm was white. At 8–13 days after flowering, the accumulated pigment in the pericarp was a light color, the thin seed coat was lighter in color than the pericarp, and the endosperm was still white. At 14–21 days after flowering, the pericarp presented dark red, the seed coat was colored but thinner than the pericarp, and the endosperm was white. Based on these observations, the pigment was mainly deposited in the pericarp, a small amount was deposited in the seed coat, and the pigment was absent in the endosperm (Figure 4B).

At 2 weeks after flowering, the pericarp and seed coat cells of the weedy rice began to shrink, and the binding between them gradually became tight. The pericarp cells began to shrink, and the pigment began to accumulate mainly in the lower part of the mesocarpal cells. A small amount of pigment was deposited in the outer pericarp. Pigments were difficult to detect in the seed coat. The aleurone cells began to form out of the outer layer of endosperm, and they arranged themselves into a rectangular shape. At 3 weeks after flowering, the cell binding between pericarp and seed coat was more compact, and the seed coat layer was completely flattened. A large amount of pigment was deposited in the pericarp, and a small amount was deposited in the seed coat. The gap between the aleurone layer and the endosperm cells shrank, and neither the aleurone layer nor the endosperm portion was pigmented (Figure 4C).

### Developmental Dynamics of Pigment Concentration and Composition in Weedy Rice Pericarp

**FIGURE 5:**
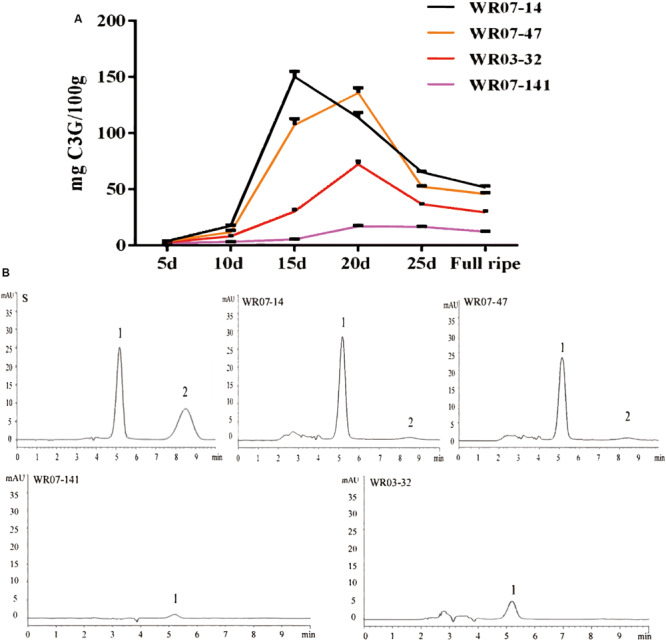
Changes in anthocyanin content in differently colored varieties of weedy rice through different developmental stages. **(A)** The four different color polylines represent the total anthocyanin content of the 4 weedy rice accessions over 30 days. **(B)** HPLC spectra showing anthocyanin types observed in 4 weedy rice accessions varieties. Peak 1 is cyanidin-3-glucoside (C3G), and peak 2 is paeonidin-3-glucoside (P3G). Graph S represents the peak positions of the standard.

To determine changes in pericarp pigmentation with weedy rice seed growth, we further measured the changes of anthocyanin concentration and composition in four typical weedy rice accessions, WR07-14, WR07-47, WR03-32, and WR07-141, since the anthocyanin is the main component of pericarp pigment. The concentration of anthocyanins rose and then fell until it stabilized, with the peak appearing sometime between the 15th to 20th day after flowering (Figure 5A). As expected, the anthocyanin concentration in dark red pericarp weedy rice WR07-14 was higher and accumulated more quickly than that in red pericarp weedy rice during the whole development period (Figure 5A). The ranking order of anthocyanin concentration and color from deep to light among rice strains was WR07-14 > WR07-47 > WR03-32 > WR07-141.

The anthocyanin components of weedy rice were detected by using the hydrochloric acid-vanillin method and high performance liquid chromatography-mass spectrometry (HPLC) (Baldi et al., 1995; Pei et al., 2017). We found that paeoniflorin (P3G) only occurred in dark red pericarp weedy rice (WR07-14 and WR07-47), based on HPLC, and proanthocyanidins only occurred in the red pericarp weedy rice (WR03-32 and WR07-141), based on the hydrochloric acid-vanillin method (Table 2). By comparing the peak positions of the 4 weedy rice accessions with the standards in the HPLC spectrum, we determined the anthocyanin composition of the samples (Figure 5B). Cyanidin (C3G) was detected in all 4 weedy rice accessions, and its concentration was higher in dark red pericarp weedy rice than in red pericarp weedy rice (Table 2).

**TABLE 2:** Anthocyanin content in different colored weedy rice varieties.

**Weedy rice variety**	**Pericarp color**	**Anthocyanin (C3G)**	**Anthocyanin (P3G)**
WR07-14	Dark red	44.92 ± 0.89^a^	5.18 ± 0.12^a^
WR07-141	Red	5.21 ± 0.22^d^	N
WR03-32	Dark red	13.9 ± 0.31^c^	N
WR07-47	Dark red	37.4 ± 0.64^b^	5.21 ± 0.29^a^

*C3G stands for cyanidin-3-glucoside and P3G stands for peonidin-3-glucoside. The different lowercase letters in the columns of the table indicate the significant difference in the content of each component between the different weedy rice at the *p* < 0.05 level. Because the color of the control cultivated rice pericarp is white and colorless, it is 0. The unit of the anthocyanins is (mg/100 g).*

### Trace Element Differences Between Weedy Rice and Cultivated Rice

Cultivated red rice is considered to be a beneficial, healthful food (Rhanissa et al., 2011). However, the functional and nutritional qualities of weedy rice in red and other colors is poorly known. The concentrations of the metal elements were measured in weedy rice accessions WR07-14, WR07-47, WR03-32, WR07-141, WR07-142 and cultivated rice accessions Shennong265, Akihikari, Nipponbare, and Qishanzhan. The concentrations of various metal elements in weedy rice were significantly higher than that of control cultivated rice, sometimes 2–3 times greater (Table 3). The concentration of Mg was higher than that of other metal elements in both weedy rice and cultivated rice, followed by Ca > Zn and Mn > Cu > Mo > Se > Ge.

**TABLE 3:** Metal element content in different rice varieties.

**Rice variety**	**Color**	**Fe**	**Ca**	**Mg**	**Mn**	**Cu**	**Zn**	**Mo**	**Se**	**Ge**
WR07-47	Dark red	42.31	261.60	765.50	43.40	3.94	129.69	0.73	0.07	0.014
WR03-32	Dark red	23.18	164.40	762.78	28.56	2.52	59.91	0.77	0.03	0.0068
WR03-29	Red	26.71	165.90	785.04	24.46	2.23	48.78	0.58	0.02	0.0008
WR07-141	Red	38.14	177.90	986.19	29.30	3.78	73.18	1.10	0.06	0.014
WR07-142	Red	20.85	127.25	1048.90	28.91	3.28	45.77	0.46	0.05	0.0069
AKI	White	9.86	55.35	458.34	11.66	1.32	16.99	0.21	0.02	0.0034
SN265	White	9.17	83.40	435.40	14.89	1.43	22.50	0.20	0.01	0.0016
QSZ	White	8.84	77.60	534.30	10.98	1.12	19.83	0.31	0.01	0.0028
NIP	White	11.37	84.00	654.60	12.87	1.44	26.01	0.22	0.02	0.0032
WR-AVE	Red	30.24^aA^	179.41^aA^	869.68^aA^	30.93^aA^	3.15^aA^	71.47^aA^	0.73^aA^	0.05^aA^	0.0085^aA^
CR-AVE	White	9.81^bB^	75.09^bB^	520.66^bB^	12.60^bB^	1.33^bB^	21.33^bB^	0.23^bB^	0.015^bB^	0.0028^bB^

*WR (Weedy rice), AKI (Akihikari), SN265 (Shennong265), QSZ (Qishanzhan), NIP (Nipponbare), WR-AVE (Weedy rice Average), CR-AVE (Cultivated Rice Average). AKI, SN265, QSZ, and NIP are control cultivated rice. The different lowercase letters in the columns of the table indicate the significant difference in the content of each component between weedy rice and control cultivated rice at *p* < 0.05 level. Capital letters indicate the significant difference in the content of each component between weedy rice and control cultivated rice at *p* < 0.01 level. The unit of the anthocyanins is (mg/kg).*

### Free Amino Acid Content Differences Between Weedy Rice and Cultivated Rice

Free amino acids are important biologically active molecules which play a role in the synthesis of proteins and in providing energy for the body and brain activity (Zarei et al., 2017). A total of 17 free essential amino acids were detected in the five weedy rice and four cultivated rice varieties analyzed (Table 4). Glutamate (Glu) was the most abundant free amino acid in both weedy and cultivated rice. The total amino acid concentration was higher in the weedy rice than in the cultivated rice, and most individual free amino acids (Asp, Glu, Val, and Thr) were significantly higher in concentration in the weedy rice than in the cultivated rice. However, Cys and Gly were significantly lower in the weedy rice than in the cultivated rice (Table 4).

**TABLE 4:** Free amino acid content in different rice varieties.

**Amino acid**	**WR07-47**	**WR03-32**	**WR03-29**	**WR07-141**	**WR07-142**	**AKI**	**SN265**	**QSZ**	**NIP**	**WR-Ave**	**CR-Ave**
Asp	7.27	5.58	5.87	6.54	6.074	4.85	4.21	4.12	3.95	6.27^aA^	4.28^bB^
Thr	2.59	1.9	2.01	2.34	2.15	1.79	1.66	1.68	1.89	2.20^aA^	1.76^bA^
Ser	3.76	2.71	2.71	2.71	2.71	2.71	2.68	2.59	2.71	2.92^aA^	2.67^aA^
Glu	13.39	12.66	10.67	12.25	11.04	9.43	9.6	9.23	9.68	12.0^aA^	9.49^bB^
Gly	3.22	2.35	2.56	2.76	2.59	3.69	3.87	3.63	3.58	2.70^bB^	3.69^aA^
Ala	4.05	2.81	3.07	3.61	3.23	3.27	3.3	3.33	3.24	3.35^aA^	3.29^aA^
Cys	2.84	2.65	2.65	2.69	2.85	3.49	3.59	3.36	3.61	2.73^bB^	3.51^aA^
Val	5.88	6.3	6.89	5.59	5.63	5.21	5.13	5.22	5.19	6.06^aA^	5.19^bA^
Met	1.96	1.612	1.793	1.82	1.78	1.8	1.83	1.78	1.67	1.79^aA^	1.77^aA^
Ile	2.28	1.49	1.78	2.13	1.89	1.65	1.63	1.75	1.55	1.91^aA^	1.65^aA^
Leu	5.03	3.702	3.77	4.62	4.077	3.63	3.67	3.55	3.48	4.24^aA^	3.58^aA^
Tyr	2.25	1.34	1.58	1.97	1.74	1.43	1.48	1.33	1.49	1.78^aA^	1.43^aA^
Phe	5.27	4.68	5.06	5.18	5.23	4.97	5.03	4.86	4.94	5.08^aA^	4.95^aA^
Lys	3.1	2.41	2.55	2.89	2.85	2.5	2.55	2.45	2.41	2.76^aA^	2.48^aA^
His	1.54	0.97	0.97	1.33	1.2	0.99	1.02	0.93	0.93	1.20^aA^	0.97^aA^
Arg	6.35	4.53	4.81	5.73	5.26	4.79	4.73	4.89	4.67	5.34^aA^	4.77^aA^
NH3	0.23	0.19	0.2	0.18	0.21	0.19	0.2	0.22	0.18	0.20^aA^	0.20^aA^
Total	71.03	57.86	59.13	64.93	60.77	56.6	55.42	56	55.4	62.64^aA^	55.87^bA^

*WR (Weedy rice), AKI (Akihikari), SN265 (Shennong265), QSZ (Qishanzhan), NIP (Nipponbare), WR-AVE (Weedy rice Average), CR-AVE (Cultivated Rice Average). AKI, SN265, QSZ, and NIP are control cultivated rice. The different lowercase letters in the columns of the table indicate the significant difference in the content of each component between weedy rice and control cultivated rice at *p* < 0.05 level. Capital letters indicate the significant difference in the content of each component between weedy rice and control cultivated rice at *p* < 0.01 level. The unit of the anthocyanins is (mg/g).*

### Fatty Acid Content Differences Among Color Pericarp Weedy Rice

Saturated fatty acids (palmitic acid and stearic acid) and unsaturated fatty acids (oleic acid and linoleic acid) were detected in weedy rice and cultivated rice in this study. We found that the overall ratio of saturated to unsaturated fatty acids in the weedy rice was slightly lower than that in cultivated rice. The ratios of saturated fatty acid and unsaturated fatty acid content were 20.53 and 79.47% in weedy rice, and the ratios in cultivated rice is 23.03 and 76.97%. There was no significant difference in the proportion of palmitic acid (a saturated fatty acid) between weedy rice and cultivated rice. The proportion of stearic acid (a saturated fatty acid) in weedy rice was significantly lower than that of control cultivated rice (Table 5). These indicators imply that weedy rice fatty acid is superior to the control cultivated rice.

**TABLE 5:** Fatty acid content in different rice varieties.

	**Saturated fatty**		**Unsaturated fatty**		
**Rice variety**	**acids**		**acids**		**Total %**
	**Palmitic acid**	**Stearic acid**	**Oleic acid**	**Linoleic acid**	
WR07-47	20.72	1.86	36.97	40.45	100.00
WR03-32	19.49	1.97	44.05	34.49	100.00
WR03-29	19.69	1.81	42.86	35.60	100.00
WR07-141	17.41	1.28	42.63	38.69	100.00
WR07-142	17.06	1.38	43.53	38.03	100.00
AKI	20.11	2.81	41.59	35.49	100.00
SN265	20.22	2.71	41.55	35.52	100.00
QSZ	20.48	2.80	41.49	35.23	100.00
NIP	20.15	2.85	41.54	35.46	100.00
WR-AVE	18.874^aA^	1.66^aA^	42.01^aA^	37.45^aA^	100.00
CR-AVE	20.24^aA^	2.79^bB^	41.54^aA^	35.42^aA^	100.00

*WR (Weedy rice), AKI (Akihikari), SN265 (Shennong265), QSZ (Qishanzhan), NIP (Nipponbare), WR-AVE (Weedy rice Average), CR-AVE (Cultivated Rice Average). AKI, SN265, QSZ, and NIP are control cultivated rice. The value represents the percentage of each fatty acid component in the total. The different lowercase letters in the columns of the table indicate the significant difference in the content of each component between weedy rice and control cultivated rice at *p* < 0.05 level. Capital letters indicate the significant difference in the content of each component between weedy rice and control cultivated rice at *p* < 0.01 level.*

## Discussion

Our research has shown certain differences in the development of pigmentation in weedy rice compared to previous studies. Liu et al. (2011) found that the growth and endosperm development of colored rice caryopsis are basically the same as those of conventional rice varieties except for pigments such as anthocyanin. Four to five days after flowering, anthocyanin began to deposit in the pericarp of the caryopsis, and then deposited in the pericarp at both ends of the caryopsis and at the vascular bundle at the back, and finally covered the entire caryopsis 10 days after flowering. The anthocyanidin in the caryopsis mainly accumulated in the 7–20 days after flowering (Liu et al., 2011). Han observed that black rice pigment was synthesized and deposited only in the pericarp of the caryopsis, and no deposition in the seed coat and aleurone layer was observed (Han et al., 2009). The anthocyanidin was deposited on the pericarp 3 days after pollination, and the anthocyanin increased rapidly after 5–6 days of pollination. After 7 days, the anthocyanids filled the entire pericarp. The far end of the caryopsis first deposited anthocyanidose, then gradually extended to the end of the embryo and stained the entire skin (Heo et al., 2006). The pigment deposition process and location of weedy rice found in the present study were basically consistent with previous studies. Pigment deposition of weedy rice has 1–2 days delay compared to cultivated rice. In our study, accumulation of pigment in weedy rice began at both ends of the caryopsis and then accumulated in the vascular bundle on the back of the caryopsis, and the entire caryopsis was covered by pigment. The time when the weedy rice pigment began to deposit was 6–8 days after flowering, and the pigmentation time of the colored rice was slightly earlier. Most pigmentation occurred slightly in seed coats.

It has been reported that anthocyanin accumulation was positively correlated with Superoxide Dismutase (SOD) in seeds, and anthocyanin accumulation led to darker seed coat color (Yi et al., 2019). In the present study, anthocyanin in grains increased with filling during the filling stage. However, the anthocyanin content decreased after the seed maturation. We speculate that the decrease in grain vigor after seed maturation leads to a decrease in anthocyanin content, specifically the decrease in self-oxidant activity leading to a rapid decline in the accumulation rate of anthocyanins. Further related mechanisms are worth to study in the future.

### Genetic Basis of Weedy Pericarp Color and Its Significance to Domestication

During the domestication of *Oryza sativa*, pericarp color was an important trait that humans targeted to improve rice quality (Qiu et al., 2017). *Oryza rufipogon* and some of the early landraces exhibited red pericarp. However, modern rice cultivars appear white due to a lack of red pigmentation in the pericarp. *Rc* and *Rd* are two key genes related to pericarp color. The wild type *Rc* allele encodes a basic helix–loop–helix (bHLH) transcription factor, and the loss-of-function mutant *rc* allele causes the change from red to white pericarp (Sweeney et al., 2006). *Rc* gene loci were shown to have contributed to rice domestication (Sweeney et al., 2007). *Rd* is another synergistic gene of rice pericarp color that enhances the effect of *Rc* gene to promote proanthocyanidin synthesis (Furukawa et al., 2007). The purple seed coat gene *Pb* and its complementary gene *Pp* are located on chromosomes 4 and 1, respectively. When the mutant allele of *Pb* is present, the pericarp is white. When the *Pb* gene is present alone, the pericarp is brown. When the genes *Pb* and *Pp* are present at the same time, the seed coat is purple (Wang et al., 2014).

In this study, the association signals of purple rice genes *Pp* and *Pb* could not be detected in red pericarp rice (weedy or cultivated) through the GWAS panel. This implies that the two genes did not contribute to the red pericarp of weedy rice because they are two rare alleles specific to purple rice. On the other hand, we found that the weedy rice may have a novel and complex genetic system of pericarp color that involves *Rc, Rd*, and other unexplored genes. Rice pericarp color was a typical domestication-related trait of cultivated rice with a relatively simple genetic basis, i.e., the *Rc* gene experienced intense artificial selection on the pericarp color. We found some minor effect or synergistic alleles that may retain an undomesticated pericarp color gene in weedy rice. Therefore, in semi-domesticated weedy rice populations, there may exist some unexplored genetic basis of different types of agronomic traits that will further clarify the process of rice domestication. For instance, a nutrient gene (*Os03g0188500*) related to glutamic acid biosynthesis is linked to a candidate pericarp color gene (*LOC_Os03g08930*), which implies a potential selection pressure related to pigments and nutrients of weedy rice.

### Significance of Weedy Rice Anthocyanin Biosynthesis

Rice anthocyanins could be a beneficial part of human diets due to their high antioxidant activities. Lei pointed out that red rice pigment was synthesized and deposited only in the pericarp of the caryopsis and absent in the seed coat and aleurone layer (Lei et al., 2006). However, a breakthrough was made by Zhu et al. who recently engineered a high-efficiency vector system for transgene stacking to enable anthocyanin biosynthesis in endosperm. They made a construct containing eight anthocyanin-related genes driven by endosperm-specific promoters and generated a novel biofortified germplasm, “Purple Endosperm Rice,” with a high anthocyanin content (Zhu et al., 2017). However, the use of such as genetically modified organism (GMO) as a staple food still requires a long process of research and approval. Alternatively, developing natural germplasm resources with high anthocyanin contents can be achieved in the short term. In particular, marker-assisted breeding can accelerate this process once the genetic basis of desired traits is determined.

Rice nutritional quality has attracted more attention in the traditional growing areas of Asia, where monotonous consumption of rice may lead to deficiencies of essential trace elements, amino acid, and other nutrients (Bouis, 2003). In this study, we found that sampled weedy rice had much greater quantities of anthocyanin, beneficial trace elements, free amino acids, and unsaturated fatty acids than the control cultivated rice. Unsaturated fats are considered healthier for consumption than saturated fats (Yasumatsu and Moritaka, 2008). Therefore, the gene resources and novel genetic systems of rice anthocyanin biosynthesis explored in this study are of great value for the development of high-anthocyanin content rice.

## Data Availability Statement

The original contributions presented in the study are included in the article/Supplementary Material, further inquiries can be directed to the corresponding authors.

## Author Contributions

DM, JS, and MZ conceived the project and experiment. JS, WW, and MZ performed the SLAF sequencing and population genetic analysis. WW observed the process of pigmentation in weedy rice and cultivated rice. WW and JyS examined the composition and content of pigment in weedy rice and cultivated rice, and analyzed the nutritional quality of weedy rice and cultivated rice. WW, JS, DM, MZ, GZ, ZL, YH, and XJ provided the germplasm and performed the germplasm management. JS and WW conducted a selection strength analysis. WW, JS, MZ, and DM interpreted the data and wrote the manuscript. All authors contributed to the manuscript and approved the submitted version.

## Conflict of Interest

The authors declare that the research was conducted in the absence of any commercial or financial relationships that could be construed as a potential conflict of interest.

## Footnotes

1 http://rice.plantbiology.msu.edu/

## Supplementary Material

FIGURE S1 QQ Plot diagram of the improved and unimproved red rice populations’ GWAS results. **(A)** The red pericarp in the sample is the result of the cultivation of rice. The lower fitting part indicates that the model is reasonable, and the upper raised part indicates that it is affected by the color-related locus of the pericarp. **(B)** The red pericarp in the sample of weedy rice. The lower fitting part indicates that the model is reasonable, and the upper raised part indicates that it is affected by the color-related locus of the pericarp.

FIGURE S2 Genome-wide selection sweep with GWAS peak. **(A)** Selection sweep plot of π ratio (π_unimproved population_/π_improved population)_, the black color line indicates the threshold line which used to determine strong or weak selection signal. **(B)** The dark green indicates selection sweep of Tajima’s *D* in unimproved population. The light green indicates selection sweep of Tajima’s *D* in improved population. The red dotted line indicates the GWAS peaks.

TABLE S1 Material information of the GWAS populations.

## Abbreviations

C3G: cyanidin-3-glucoside
GMO: genetically-modified organism
GWAS: genome-wide association study
KNN: k-nearest neighbor algorithm
P3G: paeoniflorin-3-glucoside
QTL: quantitative trait locus
WR: weedy rice
*WRAH*: weedy rice from Asian high latitudes
*WRSC*: weedy rice from south china.
