# Supplementary figures and images for: Weedy Rice as a Novel Gene Resource: A Genome-Wide Association Study of Anthocyanin Biosynthesis and an Evaluation of Nutritional Quality
Source: Front Plant Sci. 2020 Jun 11;11:878. doi: 10.3389/fpls.2020.00878 (PMC7300295; doi:10.3389/fpls.2020.00878)

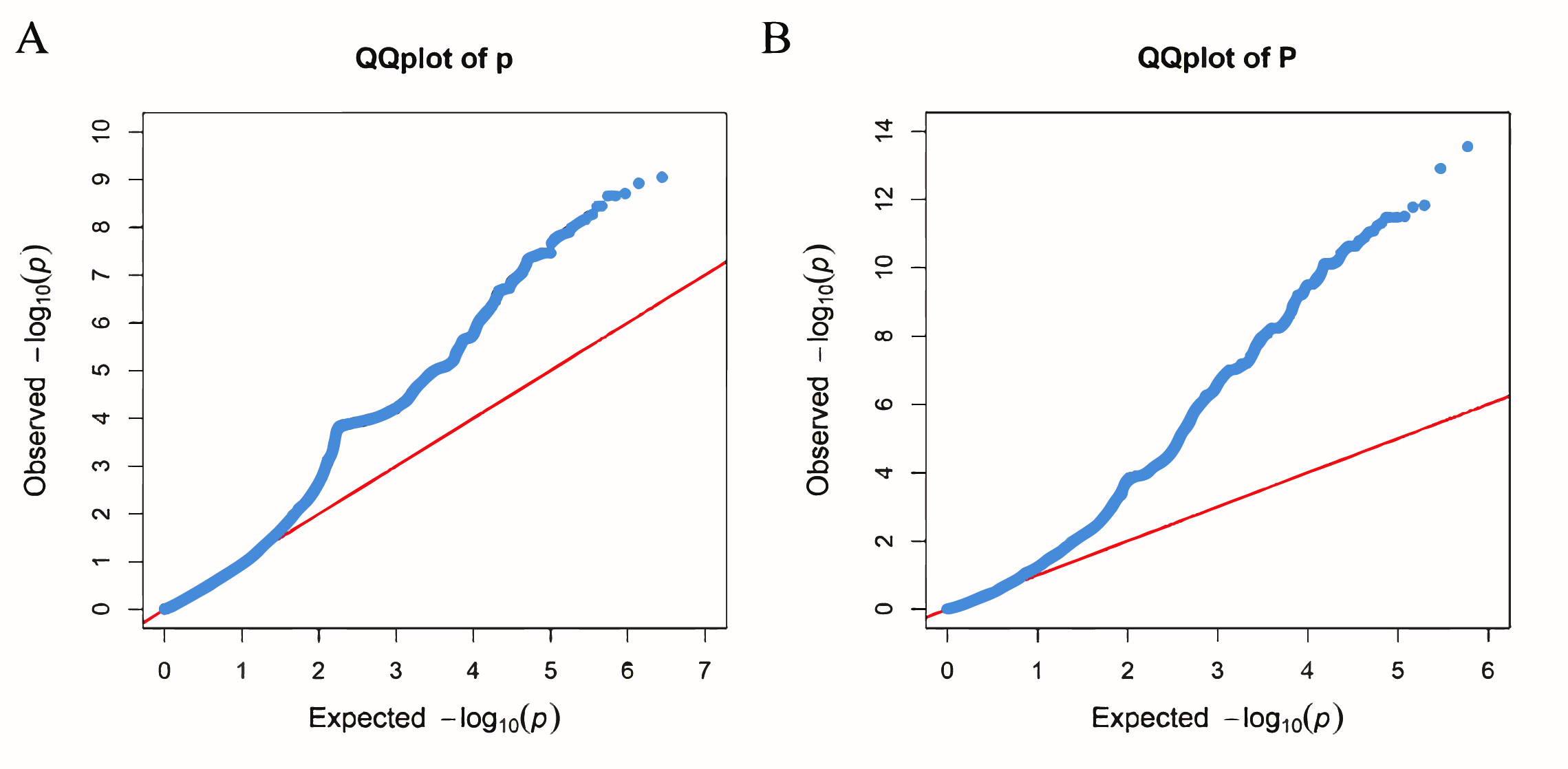

Supplement: FIGURE S1 — QQ Plot diagram of the improved and unimproved red rice populations’ GWAS results. (A) The red pericarp in the sample is the result of the cultivation of rice. The lower fitting part indicates that the model is reasonable, and the upper raised part indicates that it is affected by the color-related locus of the pericarp. (B) The red pericarp in the sample of weedy rice. The lower fitting part indicates that the model is reasonable, and the upper raised part indicates that it is affected by the color-related locus of the pericarp. [file Image_1.TIF]

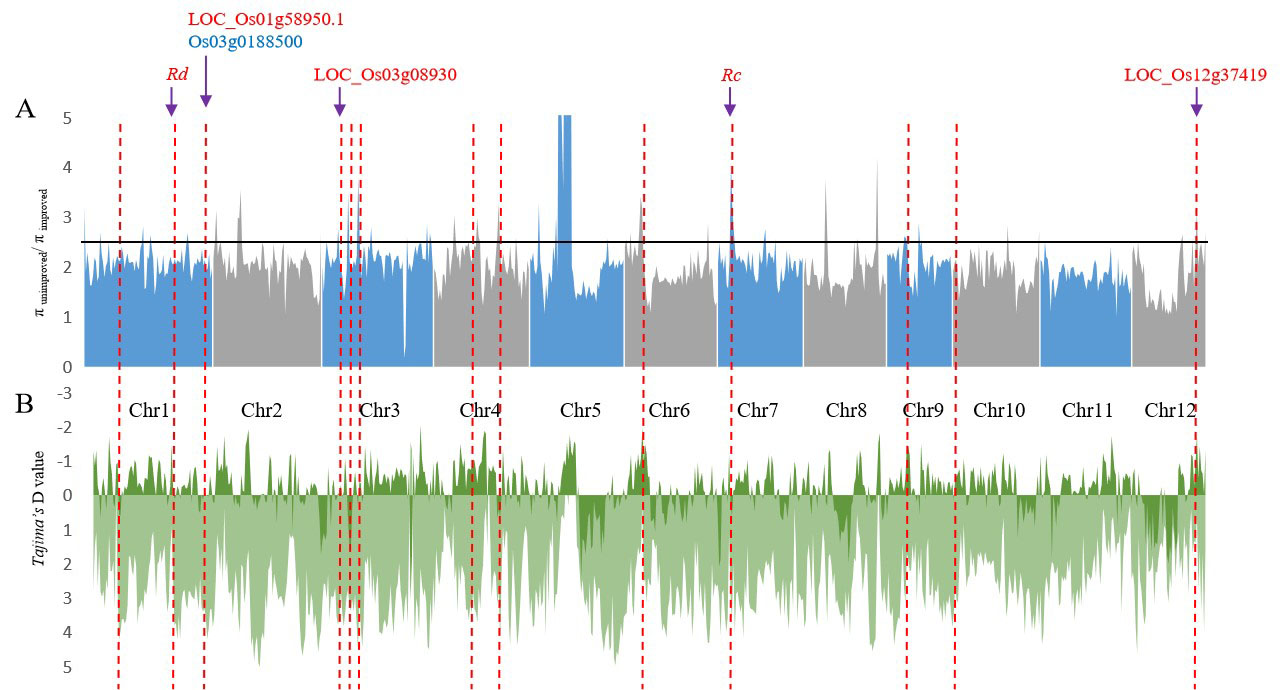

Supplement: FIGURE S2 — Genome-wide selection sweep with GWAS peak. (A) Selection sweep plot of π ratio (πunimproved population/πimproved population), the black color line indicates the threshold line which used to determine strong or weak selection signal. (B) The dark green indicates selection sweep of Tajima’s D in unimproved population. The light green indicates selection sweep of Tajima’s D in improved population. The red dotted line indicates the GWAS peaks. [file Image_2.JPEG]
